# Group-affirmation and trust in international relations: Evidence from Ukraine

**DOI:** 10.1371/journal.pone.0239944

**Published:** 2020-12-31

**Authors:** Eunbin Chung, Anna O. Pechenkina

**Affiliations:** 1 Department of Political Science, University of Utah, Salt Lake City, Utah, United States of America; 2 Department of Political Science, Utah State University, Logan, Utah, United States of America; University of Glasgow, UNITED KINGDOM

## Abstract

How can states with a history of recent armed conflict trust one another? Distrust between Ukraine and Russia aggravates security fears and limits hopes for a meaningful resolution of the bloodiest armed conflict in Europe since 1994. Hostility levels have risen dramatically between the populations of Ukraine and Russia after the events of 2013–2015. Political psychology offers two competing approaches to increase trust between the publics of different countries: appealing to an overarching, common identity above the national level vs. affirming a sense of national identity. This project asks which of these approaches increases trust towards Russia among the Ukrainian public. The study employs a survey experiment (between-subjects design) to evaluate these competing claims. The survey is to be fielded by a reputable public opinion research firm, the Kiev International Institute of Sociology, based in Ukraine.

## Introduction

Mutual animosity between Ukrainians and Russians skyrocketed as a result of recent hostilities: the mass protests in Kyiv in late 2013 to early 2014, demanding to replace a pro-Russian government with a staunchly anti-Russian one; Russia then annexed Crimea in March 2014 and fueled the Donbas War in eastern Ukraine in 2014–2015, the bloodiest armed conflict in Europe since the Bosnian War [1]. In early 2020, mutually positive opinions of Russians and Ukrainians dropped by 32–34 percentage points, while mutually negative opinions rose by 28–30 points, compared to September of 2012 (before these events) [2]. Some observers have dubbed this new hostility “the divorce” between Russia and Ukraine [3]. Given democratic politics in Ukraine, its leaders may become hostages of public opinion which views any pro-peace initiatives as appeasement of a hostile neighbor. Can social science offer any solutions to overcome this situation of distrust which inhibits conflict resolution?

This project asks what type of identity affirmation is more likely to increase trust of Ukrainians towards Russians. We draw on two competing theoretical approaches to overcoming distrust—pan-identity affirmation vs. in-group identity affirmation—to answer this question. Both approaches build on the finding that group-level identities may be affirmed just like individual-level identities [4].

## Literature review and argument

The conventional view in the literature is that a sense of belonging to an overarching, supranational group across countries generates a sense of commonness that supersedes national identities, helping reconciliation, as it downplays national differences [5–10]. In the context of Ukraine and Russia, this approach would emphasize the common East Slavic identity. In contrast to West and South Slavs, East Slavs (the pan-identity for Russians, Ukrainians, and Belarusians) are marked by both their association with the Eastern Orthodox (as opposed to Catholic) Church and the usage of the Cyrillic (as opposed to Latin) alphabet [11]. This overarching identity stems from the shared history of Kyivan Rus (882–1240), the first East Slavic state that reached its peak in the 11^th^ century [12]. After the collapse of the Soviet Union, Ukrainian and Russian historiographers tended to disagree about whether the separation of eastern Slavs into three nations began before or after the Mongol invasion of 1240 ([13], p. 125). Despite politicized attempts to usurp the legacy of Kyivan Rus, all three nations trace their origins to the Kyivan project, whose contribution was “the construction of a single identity [that] had a profound impact on the subsequent identities of all the ethnic groups that constituted the Kyivan state, […which] still forms the basis of the cultural commonalities between the three East Slavic nations” ([14], p. 2). Finally, the formation of the East Slav identity preceded the rise of the Tsardom of Russia and later the Russian empire, within which Ukrainians and Belarusians were subordinate to the Russian center. Since the East Slav identity stems from the period of pre-hierarchical relationship with Russia, it lacks the negative connotation in Ukraine.

The conventional approach therefore suggests that placing an emphasis on the easily recognizable East Slavic pan-identity can help overcome the negative effects of strong nationalisms in each country. *This approach predicts that affirming a common (Eastern Slavic) identity tends to increase trust*.

Hypothesis 1: Individuals, whose *overarching*, *superordinate identity* was affirmed, exhibit more trust toward outer group than non-affirmed individuals and individuals with affirmed national identity.

In contrast, a challenging view argues that affirmation of national identities increases trust between groups [15, 16]. This new approach draws on a set of findings from the psychology of group attachment: in-group affinity does not require hostility toward other groups [17, 18]. That is, the affirmation of national identity is conceptually distinct from chauvinistic appeals to national superiority. This idea is consistent with the theoretical foundation of self-affirmation theory that those who have a clear, secure, and content sense of self tend to be more open, evenhanded, and less defensive toward others [19]. Affirming one’s own identity has thus been found to increase trust between groups across partisan [20] and racial lines [21], from the perspective of both minority groups [22] and the dominant class [23] In international relations, if each national population reflects upon the values of their national identity, trust can increase between countries. In the context of Ukraine, this approach would emphasize the national identity of Ukrainians without any allusions or comparisons to Russia. *This approach predicts that affirming a national (Ukrainian) identity tends to increase trust*.

Hypothesis 2: Individuals, whose *national identity* was affirmed, exhibit more trust toward outer group than non-affirmed individuals and individuals with affirmed overarching identity.

Whether societies can overcome trauma that stems from armed conflict impacts the stability of international interactions among states. This research will help explain how post-conflict societies can overcome distrust of former adversaries, a question of both academic and policy interest.

## Materials and methods (research design)

### Survey instrument–experimental conditions

The University of Utah Institutional Review Board approved this study (Approval number IRB_00131638). Consent will be obtained via telephone when the Kiev International Institute of Sociology fields the survey. To test our hypotheses, we designed a survey experiment with two treatments and a control group, such that 1/3 of the sample will receive the group-affirmation of the overarching Eastern Slavic identity; 1/3—will receive the group-affirmation of the national Ukrainian identity; 1/3—will serve as a control that follows the same structure as treatment but does not allude to identity, instead asking about dessert preferences (between-subjects design). The two treatments serve as independent variables of the study.

See these questions below:

### Treatment 1: Eastern Slavic identity affirmation:

1. There are many positive aspects about being Eastern Slavic. Please choose only one of the following items that you think is the most important value for Eastern Slavs:
  - family; appearance / fashion; patience; working hard; political liberty / democracy
2. Why did you choose the value you chose above as the most important to Eastern Slavs? Why do you think that value is important to Eastern Slavs? Please explain your choice in 1–2 sentences.
  - Open answer
3. How is the value you chose above expressed among Eastern Slavs? Please answer in 1–2 sentences or give an example
  - Open answer
4. Manipulation check: The task on values made me think about:
  - Things Eastern Slavs value about themselves / Things Eastern Slavs do NOT value about themselves.

This manipulation check is a simple yet straightforward way of verifying whether participants were paying attention to the task and thus thinking about values associated with said identity. In addition, this measure has been used as a reliable manipulation check in previous research that uses experimental treatments of identity affirmation [24].

### Treatment 2: Ukrainian identity affirmation

This treatment is identical to treatment 1, except all instances of “Eastern Slavic or Eastern Slavs” are substituted with “Ukrainian.”

### Control

1. Marmeladki (jellybeans) are a chewy candy. The following is a list of flavors of marmeladki. Please choose only one of the following flavors that you think will be tastiest.
  - Sizzling Cinnamon / Tropical Mango / Apple Jack / Blueberry Balloon / Tutti-Fruitti
2. Please explain why you think the marmeladki (jellybeans) you chose will be tastiest in 1–2 sentences.
  - Open answer
3. When you imagine the taste of the marmeladki (jellybeans) you chose, what do you think it would taste like compared to the others you did not choose? Please explain your choice in 1–2 sentences.
  - Open answer
4. Manipulation check: The task on jellybeans made me think about:
  - Flavors I would like / Flavors I would NOT like

Since respondents will be randomly assigned to each of the three groups, these groups will be comparable on average on both observable and unobservable characteristics. Except for the type of identity (Eastern Slavic or Ukrainian), treatments 1 and 2 are identical. The control group follows a similar procedure but does not consider anything related to identity. Thus, any intergroup differences in trust towards Russian government/people should be attributable to the treatment.

The control task on jellybeans is borrowed from earlier psychological experiments that test the impacts of identity affirmation [25]. This control task compares favorably to alternatives used in other studies. For instance, some control conditions ask respondents to think about values that are NOT important to their identities. Such a control is unlikely to serve its intended function, since the very idea of considering values that respondents deem unimportant directly invokes their evaluation of values relevant to the specified identity. It is crucial in the design of the study that the control task is similar in structure to the treatment, however is substantively unrelated to treatment (in our case, group values associated with group identities). In summary, the control task on dessert/candy preferences is substantively irrelevant to treatment conditions, yet mimics the structure of the treatment exercise.

We will use the control measures (described below) to ensure that randomization indeed delivered on average comparable groups of respondents.

### Survey instrument–dependent variables

Four questions will record the dependent variables (all responses are recorded on a 5-item scale): i) how much respondents trust the Russian government; ii) whether respondents believe that the Russian government would exploit Ukraine for its own benefit, or treat Ukraine fairly; iii) how much respondents trust the Russian people; iv) how selfish or kind the respondents believe that the Russian people are. These trust questions are adapted from the general trust questions in the World Values Survey.

In addition to using the original four dependent measures, we will add (if appropriate, as explained below) three additional composite measures:

- trust towards the Russian government, a combination of items i) and ii),
- trust towards the Russian people, a combination of items iii) and iv),
- overall trust towards Russia, a combination of items i) through iv).

To create these three indexes, we will calculate the scale reliability coefficient (Cronbach’s alpha) for the indicated items (standardized Cronbach’s alpha statistics (continuous measures bounded between 0 and 1).

Researchers using Cronbach’s alpha should be aware of the number of constituent items, as more than six will yield a high alpha even with low interitem correlation ([26], p. 102–103). This is not a problem in our case, as 2 and 4 items will be combined at a time. Given how few items are utilized, we will consider alpha above 0.6 as sufficiently high.

Additionally, Cronbach’s alpha assumes unidimensionality ([26], p. 102–103), thus one should verify whether the items all measure the same underlying component. To verify this, we will perform principal component analysis and report whether the four dependent measures—designed to capture a single underlying concept of trust—indeed all load on a single factor or at least whether there is a single factor within pairs of items i)-ii) and iii)-iv). If we identify more than one factor, then the usage of Cronbach’s alpha is inappropriate and we will need to reevaluate the validity of the dependent measures.

In the case that the interitem correlation is not sufficiently high or if the items are not unidimensional, we will abstain from using index measures and will only use the original four dependent variables.

Finally, as an additional set of analyses, we will report the results for the dependent measures that include “don’t know” responses modeled as a separate answer category.

### Survey instrument–controls

The survey will collect relevant information (about sex, age, education, ethnic group and place of residence, main occupation, income, language, political preferences), and other attributes to ensure that randomization was done properly.

### Sample, data inclusion criteria, and power analysis

No pilot data has been collected for this study. The survey will be fielded by the Kiev International Institute of Sociology (KIIS) in Ukraine in late May-June 2020 as a phone interview with a random sample of 2,000 individuals. The survey is described as a study on public opinion, and includes participants in the study if they are over 18 years of age. The KIIS obtains a verbal confirmation that respondents:

- understand the risks and benefits of participation (prior to that, the interviewer will have explained that while there are no direct benefits to the respondents, this research will increase our knowledge of public opinion in Ukraine),
- have asked any questions they might have, and
- understand how to stop their participation in the study if they choose to do so (prior to that, the interviewer will have explained that respondents may stop the interview at any point, however they cannot withdraw their participation after the interview ends, because the records are fully anonymous and it is impossible to tell which data are whose).

A respondent’s verbal yes to these three items constitutes providing informed consent at the beginning of the survey.

The KIIS uses software to generate random mobile telephone numbers; after removing non-existing phone numbers, 2,000 phone numbers are randomly selected and contacted. The rate of mobile phone ownership in Ukraine is 96% among adults; furthermore, only 7% of respondents reported that they regularly use a landline phone, and only 1% of respondents reported no access to a mobile phone. These statistics are based on survey results obtained by the KIIS team face-to-face in February 2020.

The KIIS staff are not able to perform randomization by strata in telephone interviews. We will use the demographic attributes to determine whether the groups are comparable on average. The KIIS will also reweigh the sample based on four attributes (macroregions, type of settlement, age, gender) in accordance with 2019 data collected by the Central Election Commission of Ukraine and the State Statistics Service of Ukraine; these weights will be used in the regression analyses.

The power analysis was conducted using Soper’s [27, 28] software. For the desired statistical power level of 0.8, the probability level of 0.05, for 10 predictors (treatment, baseline attitudes towards Russia, ideology, plus a battery of demographic indicators), the required sample size per group should be between 333 and 549 respondents to discern the anticipated effect size of between 0.05 and 0.03 respectively (the effect size range is based on previous studies by Chung [15], Chung and Woo [16]).

We also plan to use t-tests for group-level differences. If the effect size is very small (Cohen’s d = 0.1), then we would need 1,238 respondents per treatment to discern such an effect (while we are likely to have no more than 400–600 individuals). However, if Cohen’s d = 0.2, then our sample size would be sufficient, as only 310 respondents per treatment would be required to uncover such an effect (we describe the steps in the planned analyses in Table 1).

**Table 1:** Design planner for the study “group-affirmation and trust in international relations”.

Question	Hypothesis	Sampling plan	Analysis plan	Interpretation given different outcomes
What level of identity affirmation increases trust between states after armed conflict?	H1: Affirmation of *pan-identity* increases trust towards adversary	Random sample of landline phone numbers in Ukraine using quotas for age and geographic location. At least 600 respondents per condition. Total of 2,000 respondents.	1. Remove C/IE units.	
			2. Conduct analysis of the manipulation checks.	
			3. Recalculate power of the test: do we have enough units to maintain the power of 0.8?	If we have fewer than 333–549 respondents per group, then the power of the test is 0.8 to uncover the effect size of 0.05 to 0.03 percentage points respectively.
			4. Use demographic data to ensure treatment is random with respect to age, gender, education, income, location.	Two-tail two-sample t-tests are expected to yield substantively and statistically negligible (p-value of less than 0.05) differences-in-means between 3 treatment groups.
			5. Visualization of trust levels by treatment group.	
			6. Group comparisons via differences-in-means of average trust levels between treatment and control groups.	Calculate Cohen’s d and determine the effect size. Determine whether the sample has at least 0.8 power to detect an effect.
				If the one-tail two-sample t-tests yield that the proportion of trusting respondents is at least 2–5 percentage points higher (and is statistically discernible, i.e., p-value of less than 0.05) in the treatment 1 group, relative to the control group and to the treatment 2 group, then such an outcome will be consistent with hypothesis 1.
				If the t-test uncovers a statistically discernible but substantively smaller effect size, or if the t-test uncovers an expected effect size that is statistically negligible, we will conclude that there is no evidence of a difference between treatments. Discuss whether the test had enough power to detect an effect.
			7. Individual-level determinants of trust via the multivariate ordered logistic regressions:	The hypotheses expect that the binary indicators of treatment will have a positive and statistically discernible association with the continuous measures of trust (coded from low to high level). We will compare the direction and the marginal effect of IVs on both DVs.
			• - DV1 = continuous measure of trust toward Russian government.	
			• - DV2 = continuous measure of trust toward Russian people.	If treatment 1 indicator produces changes in the DV in the opposite direction or changes that cannot be distinguished from the control group, then we will take that as no evidence of difference between treatment 1 and control.
			• - IVs = binary indicators of treatment 1 or treatment 2 (control is the baseline)	
			• - Controls: demographics, prior attitudes toward Russia, ideology.	If treatment 1 indicator produces changes in the DV in the expected direction and the effect is statistically distinguishable from the control group, then we will take that as evidence consistent with hypothesis 1 and will focus on the effect size as described below.
				If both indicators produce positive changes in the DVs and both are statistically discernible, but the effect size of treatment 1 is larger, we will discuss this as evidence that pan-identity affirmation generates higher levels of trust than the affirmation of national identity (consistent with H1).
	H2: Affirmation of *national* identity increases trust towards adversary		Steps 1–5 of the analysis plan and interpretation are repeated.	
			6. Group comparisons via differences-in-means of average trust levels between treatment groups.	Calculate Cohen’s d and determine the effect size. Determine whether the sample has at least 0.8 power to detect an effect.
				If the one-tail two-sample t-tests yield that the proportion of trusting respondents in treatment 2 is at least 2–5 percentage points higher (and is statistically discernible, i.e., p-value of less than 0.05), relative to the control group and to the treatment 1 group, then such an outcome will be consistent with hypothesis 2.
				If the t-test uncovers a statistically discernible but substantively smaller effect size or if the t-test uncovers the expected effect size that is statistically negligible, we will conclude that there is no evidence of a difference between treatments.
				Discuss whether the test had enough power to detect an effect
			7. Individual-level determinants of trust via the multivariate ordered logistic regressions:	If treatment 2 indicator produces changes in the DV in the opposite direction or changes that cannot be distinguished from the control group, then we will take that as no evidence of difference between treatment 2 and control.
			• - DV1 = continuous measure of trust toward Russian government.	
			• - DV2 = continuous measure of trust toward Russian people.	If treatment 2 indicator produces changes in the DV in the expected direction and the effect is statistically distinguishable from the control group, then we will take that as evidence consistent with hypothesis 2 and will focus on the effect size as described below.
			• - IVs = binary indicators of treatment 1 or treatment 2 (control is the baseline)	
			• - Controls: demographics, prior attitudes toward Russia, ideology.	If both indicators produce positive changes in the DVs and both are statistically discernible, but the effect size of treatment 2 is larger, we will discuss this as evidence that national identity affirmation generates higher levels of trust than the pan-identity affirmation (consistent with H2).

The sample of 2,000 will be randomly divided into 3 groups of 600–740 individuals each; in phone surveys, the KIIS may only conduct randomization during the survey, so the exact size of each group cannot be predetermined.

### Data exclusion criteria and terminating data collection

The data collection will terminate after a sufficient number of complete survey units are acquired (2,000). At the data collection stage, the KIIS will only take into account whether respondents completed all survey items. Data will be made available upon study completion.

To remove ‘careless or insufficient effort (C/IE)’ responses in surveys, Curran & College [29] suggest a combination of screening for extreme response times and ‘long-string analysis’ [30, 31], which examines the longest string of identical responses. Response time screenings are less relevant in this study as we are employing a phone survey and interviewers will need to take the time to read questions and record responses.

However, it is possible respondents might try to rush through the survey by, for example, absentmindedly choosing the first response option for a series of questions. Therefore, data will be *excluded on the basis of ‘long-string analysis’*. Such responses will be calculated based on the response option that is selected most frequently for each participant. Following the baseline rule of thumb of a conservative cut score suggested by Curran & College ([29], p.16), we will consider individuals with a string of consistent responses equal to or greater than half of the length of the total scale as C/IE responders. That is, as almost all of our response options are presented on 5-point Likert scales, individuals who deliver a string of the same response to three or four consecutive Likert items will be removed. For robustness, we will replicate our results first excluding three consecutive identical responses, then excluding four identical consecutive responses.

To maintain the power of the test at 0.8, our sample of 2,000 participants allows for at least 50–179 (depending on the effect size) listwise deletions of units that do meet the criteria for exclusion as outlined here.

### Positive controls

The survey includes two manipulation checks to measure whether the treatments operate as intended. First, after each treatment, respondents are asked whether the task on values asked them to think about things Eastern Slavs (in treatment 1) or Ukrainians (in treatment 2) value about themselves and do not value about themselves. In the control group, the check asks whether the task on jellybeans made them think about flavors they think will be tasty or not tasty. These are tasks that have been used in psychological experiments testing identity affirmation [25]. If we observe few/no respondents selecting the latter option, our confidence that respondents are engaging with the survey and the treatments will increase.

Second, half the sample is asked to self-report their attachment to the Ukrainian and Eastern Slavic identity before treatments are administered, while the other half is asked to do so after the treatments. We will calculate the difference between ‘before’ and ‘after’ groups within treatment 1, within treatment 2, and within control group. If we observe an increase in the ‘after’ groups in treatments 1 and 2, but not in the control group, our confidence that the treatments actually manipulate one’s attachment to these identities will increase.

### Inference

We will observe overall proportions of positive answers to the trust questions by treatment, as well as create scatter plots of intensity of trust by treatment.

In addition, we will conduct pairwise two-tail two-sample t-tests to estimate whether there are systematic average between-group differences in trust levels (between the three groups of respondents).

Finally, to understand the individual-level determinants of trust, multivariate analysis will be estimated via ordinary least squares regression models. The dependent variable is a continuous index between 0 and 1 of how much trust a respondent exhibits towards Russia, calculated as standardized Cochrane’s alpha statistic based on the interitem correlation of two items that capture one’s trust towards the Russian government and towards Russian people. The predictors will include experimental conditions, ideology, and demographic attributes. The criteria for statistical inference will follow a frequentist framework with cutoff levels of statistical discernibility of 0.05.

We have registered the experimental design and procedures described in this report on OSF or AsPredicted [32].
