# Correction: Group-affirmation and trust in international relations: Evidence from Ukraine

**DOI:** 10.1371/journal.pone.0275165

**Published:** 2022-09-20

**Authors:** The PLOS ONE Editors

This article [1] did not meet the journal’s requirements for Registered Report Protocols; it was submitted to *PLOS ONE* before the study began, but participant recruitment and/or data collection began before the protocol was accepted for publication.

Therefore, this article should be considered by readers as a peer-reviewed Study Protocol rather than a Registered Report Protocol.

We regret that this issue was not identified prior to publication.

## References

[1] Chung E, Pechenkina AO (2020) Group-affirmation and trust in international relations: Evidence from Ukraine. PLoS ONE 15(12): e0239944. 10.1371/journal.pone.0239944 33382719 PMC7774829

